# Progress towards a 256 channel multi-anode microchannel plate photomultiplier system with picosecond timing

**DOI:** 10.1016/j.nima.2011.11.019

**Published:** 2012-12-11

**Authors:** J.S. Lapington, T.J.R. Ashton, D. Ross, T. Conneely

**Affiliations:** a Space Research Centre, University of Leicester, UK; b Photek Ltd., St. Leonards-on-sea, East Sussex, UK

**Keywords:** microchannel plate, photon counting detector, picosecond timing, multi-channel, fluorescence lifetime imaging, fluorescence correlation spectroscopy, high content, single molecule imaging

## Abstract

Despite the rapid advances in solid state technologies such as the silicon photomultiplier (SiPM), microchannel plate (MCP) photomultipliers still offer a proven and practical technological solution for high channel count pixellated photon-counting systems with very high time resolution. We describe progress towards a 256 channel optical photon-counting system using CERN-developed NINO and HTDC ASICs, and designed primarily for time resolved spectroscopy in life science applications.

Having previously built and demonstrated a 18 mm diameter prototype tube with an 8×8 channel readout configuration and <43 ps rms single photon timing resolution, we are currently developing a 40 mm device with a 32×32 channel readout. Initially this will be populated with a 256 channel electronics system comprising four sets of modular 64 channel preamplifier/discriminator, and time-to-digital converter units, arranged in a compact three dimensional configuration.

We describe the detector and electronics design and operation, and present performance measurements from the 256 channel development system. We discuss enhancements to the system including higher channel count and the use of application specific on-board signal processing capabilities.

## 1 Introduction

This work is being carried out within a project called “Information Rich Imaging of Cells” (IRPICS) and is funded through the BBSRC Technology Development Research Initiative scheme. The hardware goal of the project is to develop a high throughput multi-channel photomultiplier with picosecond event timing for high content imaging in life science applications using time resolved spectroscopy. IRPICS has built on the achievements of the HiContent project [1,2], an STFC funded proof-of-principle demonstration of a small pore microchannel plate photomultiplier with a discrete pixel readout. Each pixel is read out using high speed multi-channel electronics comprising ASICs developed at CERN for the LHC experiment. The goals for IRPICS are to increase the channel count and improve throughput, to integrate detector and electronics into a viable system to interface with a standard optical microscope as a replacement for a commercial camera, and to have a digital processing system capable of being reprogrammed for a range of applications e.g. fluorescence lifetime imaging, fluorescence correlation spectroscopy, etc. using techniques such as Forster resonance energy transfer.

One of the primary applications at which IRPICS is aimed is time correlated single photon counting (TCSPC). In conventional single channel TCSPC, illumination intensity must be maintained sufficiently low to ensure that the probability of a second fluorescence photon occurring within the dead time of the detector is negligible; otherwise the preferential loss of late photons that this causes distorts the decay time calculation. This limitation is mitigated in multi-channel devices since the second photon may be detected in a parallel channel and not lost [3]. IRPICS, having up to 1024 channels is expected to allow an increase in illumination intensity of several orders of magnitude compared to conventional TCSPC operation.

## 2 Detector design

The IRPICS detector upper mechanical design, comprising photocathode and microchannel plate, is very similar to that used for HiContent, apart from the larger MCP pore diameter (5 μm as opposed to 3 μm), which is required because of the unavailability of the smaller pore size in the larger MCP sizes.

**Fig. 1:**
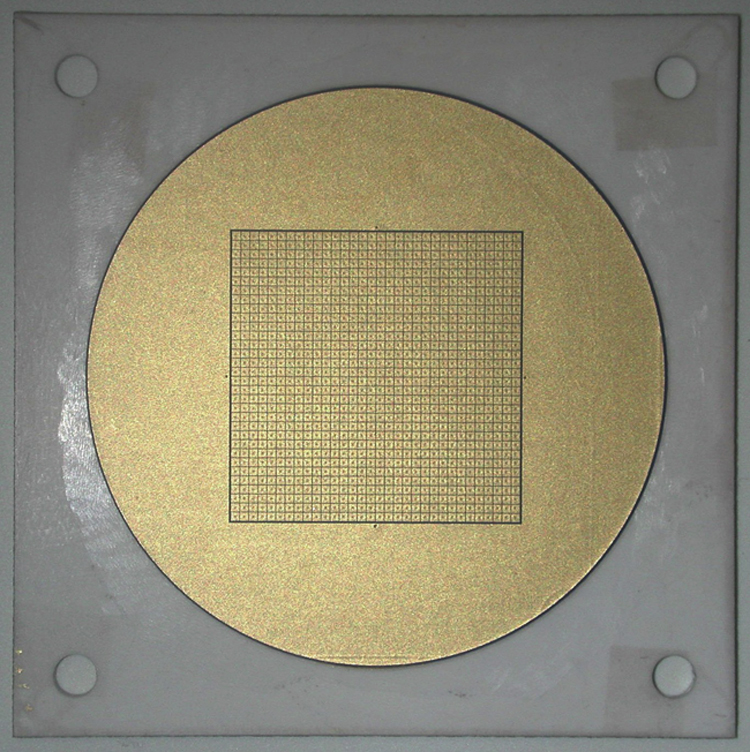
A photograph of the inside face of the IRPICS MLC readout. The 32 × 32 pixel^2^ array is surrounded by a guard anode. The detector flange is brazed on to the unmetallized surface outside the guard anode diameter (photo courtesy of Antonio Teixeira, CERN).

A multilayer ceramic (MLC) provides the pixellated readout and acts as the rear wall of the vacuum tube, incorporating 1028 electrical vacuum feed-throughs. The same MLC manufacturing technology used by CERN for the HiContent 8×8 readout is being used. However the detector size has increased 18 mm to 40 mm and the pixel format from 8×8 pixel^2^ to 32×32 pixel^2^, and the pitch reduced from 1.6 mm to 0.88 mm, almost quadrupling the geometric channel density. Fig. 1 shows a photo of the detecting surface of the 32×32 pixel^2^ MLC. Each pad is connected by a via to the corresponding pad on the opposite surface. Four additional vias and pads provide connection for the surrounding guard anode. Owing to the higher force anticipated for spring-loaded electrical connections, the method of integration with the detector body design was changed from HiContent. The MLC now forms the detector base so that the clamping force is transmitted directly through the ceramic, as opposed to the previous arrangement where it was transmitted through a kovar flange brazed to the ceramic.

**Fig. 2:**
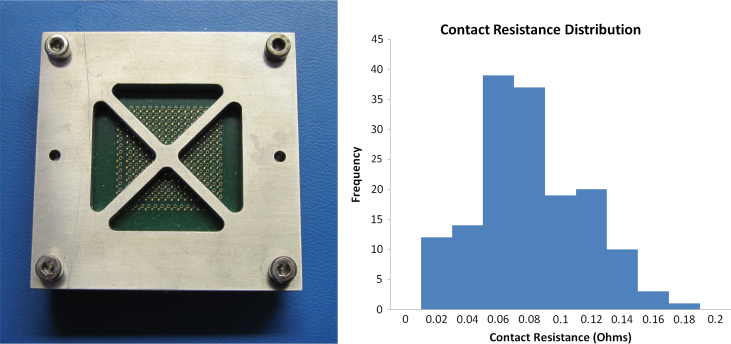
A photograph of the test fixture used to measure the contact resistance using an anisotropic conductive film interposed between representatively sized pads on two PCBs clamped together. The histogram shows the distribution of resistances for 155 contacts.

The HiContent prototype detector, with its relatively coarse 8×8 pixel format at 1.6 mm pixel pitch, utilized a pogo-pin array to provide electrical contact for the readout. This proved problematic to assemble and somewhat unreliable even with only 64 contacts. The baseline solution for the 32×32 pixel^2^ IRPICS readout anode (all connections must be made irrespective of channel aggregation later in the chain) was to use a Land Grid Array (LGA) socket technique using miniature spring-loaded pins. While this is used routinely for chip to board interconnects, the custom devices for which we were quoted required a compression of several 10's of kilograms for our 1028 pin MLC, and the mechanical complications that this generated led us to investigate alternative, demountable methods. Various types of anisotropic conductive films (ACF) have been developed recently. We have selected, tested and proven a particular ACF material, manufactured by Shin-Etsu Polymer Co. Ltd. of type MT–P [4]. This ACF comprises a regular array of plated wires at a pitch of 0.1 mm embedded in and slightly projecting from, a sheet of silicone rubber 0.25 mm thick. This is clamped between the two mating contact surfaces, which have geometrically matching pad arrays. We manufactured PCBs with representative pad sizes and a simple jig to test the consistency and reliability of the ACF material. The pad size was 0.4 mm and solder balls were manufactured on one set of pads to increase the local pressure and ACF deformation and promote contact. A photo of the test jig and results showing the consistency of contact resistance of the 155 contacts measured are shown in Fig. 2.

## 3 System design

Since the IRPICS system was primarily designed to be fitted to a typical optical microscope for use in biological research e.g. fluorescence lifetime imaging, we have integrated and miniaturized the system into a format so that it can easily be retro-fitted in place of a commercial camera system. This requirement has driven the system dimensions and choice of detector mount. The completed has dimensions of 100×100×150 mm^3^ approx. and utilizes a Canon C-mount for the microscope interface.

Though the IRPICS detector has been designed with a 1024 pixel readout, arranged in a 32×32 format, we have chosen initially to multiplex the outputs into groups of 2×2 pixel^2^ per electronics channel. The 256 channels are instrumented with four sets of modular 64 channel pulse processing chains. The detector mounts on a headboard, which distributes the signals to the processing chains, each of which comprises a front-end board closely coupled to a time-to-digital (TDC) convertor board. The front-end board houses two 32 channel NINO ASICs [5], which are custom designed for this project. They are a low power, higher channel modification of the original 8 channel NINO ASIC [6], a multi-channel preamplifier/discriminator chip. Each modular TDC board utilizes two 32 channel HPTDC ASICs [7]. Both ASICs were originally designed for the RPC Trigger detectors on the LHC ALICE experiment. The outputs of each processing chain interface to a backplane, which itself interfaces to the FPGA-based digital processing board. The reprogrammability of the FPGA allows different modes of operation to be accommodated without change of hardware. The digital processor communicates via a USB 2.0 interface to a PC for FPGA programming, software instrument control and data downlink.

**Fig. 3:**
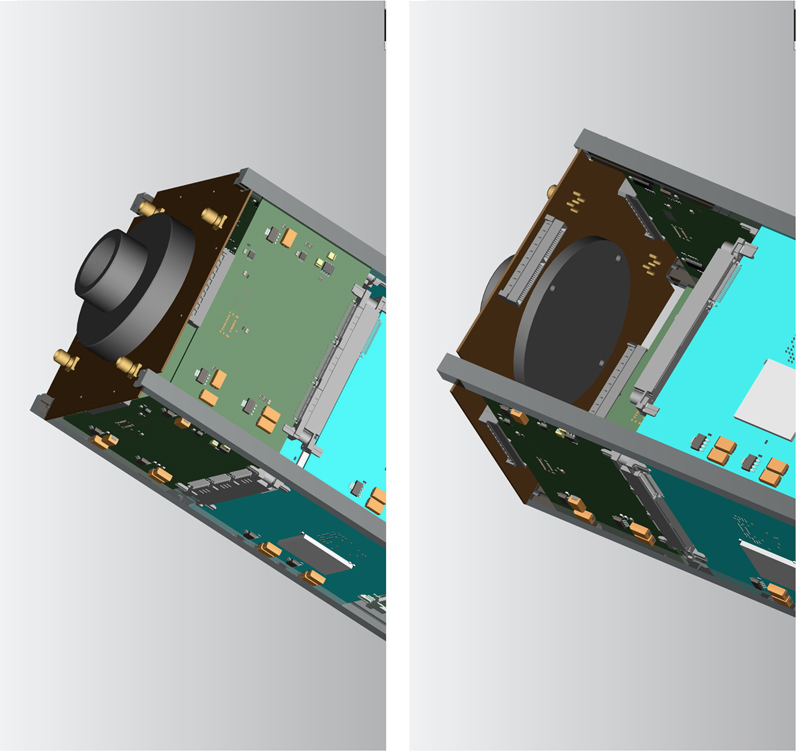
Two views from a 3D CAD drawing of the detector and electronics system. The detector mounts on the passive headboard which distributes the 256 signals to four 64 channel NINO preamplifier/discriminator boards on four sides of the square cross-section. Each NINO board mates with a 64 channel HPTDC board. The backplane and digital processing board are to the right but not visible.

The CAD drawings in Fig. 3 illustrate the three dimensional configuration of the electronics boards. The detector (an 18 mm version shown here) is the black, stepped cylinder mounted on the detector headboard (brown), its window facing outwards. The detector headboard attaches via 4 edge connectors to four 64 channel pre-amplifier boards (green), which in turn attach via impedance-matched connectors to the four TDC boards (turquoise). This integrated arrangement has been chosen to provide a design as compact as possible, in order to mount the system on a microscope in place of a typical CCD camera head.

## 4 Electronics design and measurements

**Fig. 4:**
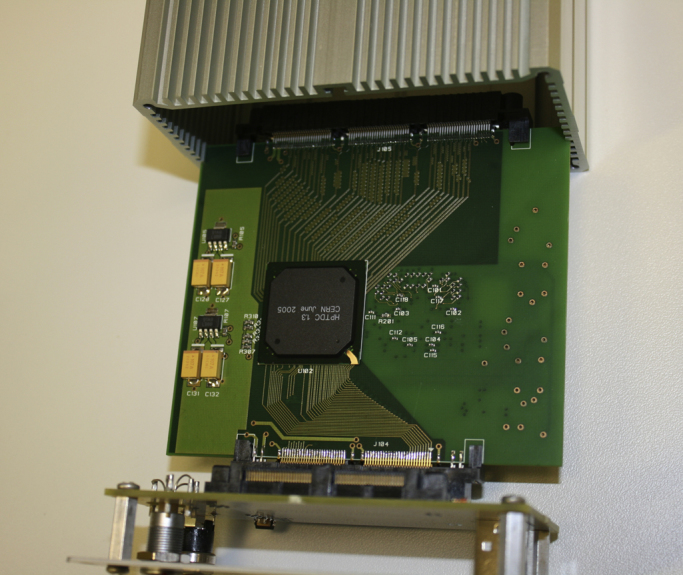
A photograph of the modular 64 channel TDC board mounted on a backplane (in this case designed for a single TDC board). This board houses HPTDC ASICs; a second HPTDC is located on its underside.

**Fig. 5:**
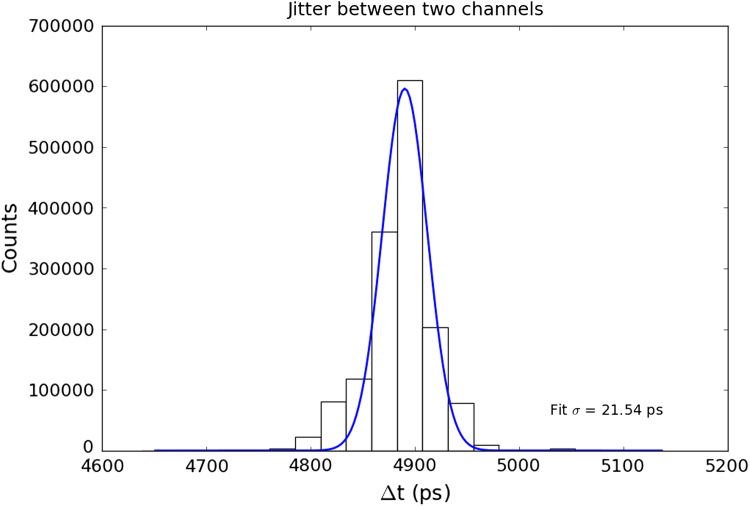
A measurement of the time jitter between 2 channels of the IRPICS modular TDC board using an electronically generated signal to feed both inputs simultaneously. The standard deviation of the measured time jitter is 21.54 ps.

The modular electronics are currently in varying states of readiness. The NINO32 chip has already been thoroughly bench tested and results published [5], however the modular front-end board, which incorporates this in IRPICS is only just being manufactured. The backend has progressed further. The modular TDC board has been designed, manufactured and tested, and is shown in Fig. 4. Preliminary measurements of the channel to channel time jitter using an electronically generated test pulse are very promising; a standard deviation of 21.54 ps has been measured and the data is shown in Fig. 5. To put this figure in perspective, the time bin width in the HPTDC ASIC “very high resolution” mode is 24.41 ps.

Fig. 5 is a photo of the modular TDC board. The 64 channel NINO front-end board interfaces via the front edge connector visible in the lower left hand corner. This photo shows an alternative backplane design for a single TDC board (available from Photek as a standalone 64 channel TDC system). The backplane also interfaces (via the upper visible unpopulated connector) with the FPGA-based digital processing board.

**Fig. 6:**
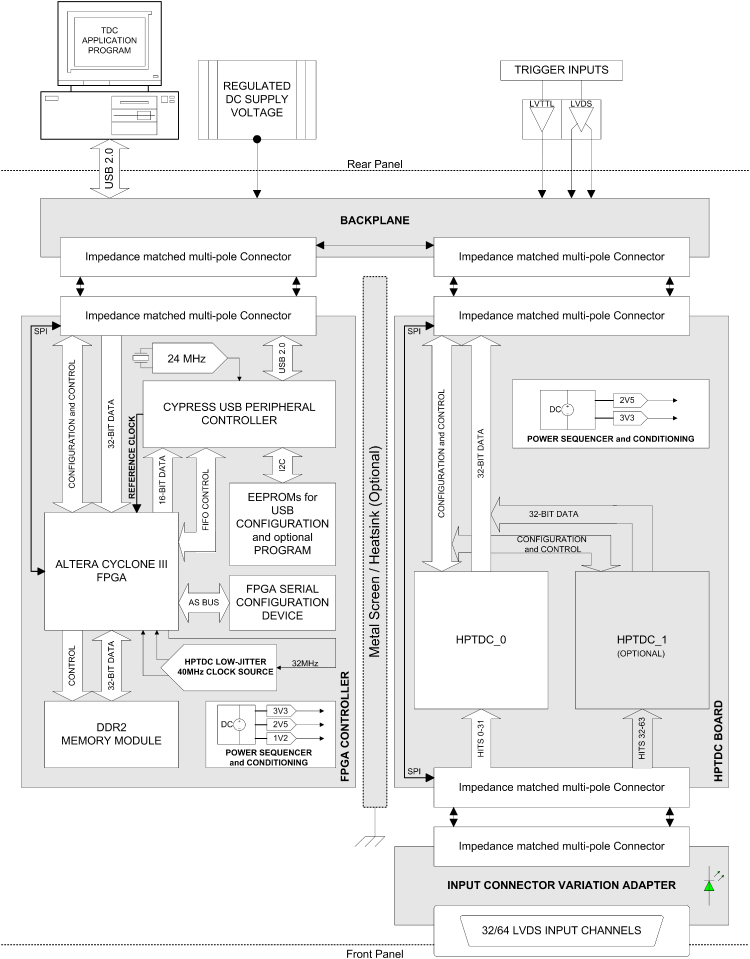
A figure showing a block diagram of the digital processing elements of the detector electronics (the NINO pre-amplifier board attaches in place of the input connector variation adapter shown).

Fig. 6 is a block diagram of the FPGA controller board (left), connected with one 64 channel TDC board (right). The remaining 3 TDC boards required for the 256 channel system have been omitted for clarity. The Altera FPGA can be easily reconfigured to apply various application specific firmwares to suit the required mode of operation. Pre-processing in the FPGA is used to provide data compression and analysis functionality upstream of the USB 2.0 PC interface, which is limited to –200 Mbit/s, and would otherwise constitute a data throughput bottleneck. The on-board RAM, shown in the diagram but not implemented in the initial build, will be added to later versions of the board to provide burst mode data acquisition at speeds limited only by the detector and pulse processing chain capabilities. The FPGA undertakes several tasks; it configures the TDCs on boot-up, controls the data acquisition, and performs pre-processing on TDC data such as implementing INL correction, filtering out unnecessary data attributes, and converting data from an event stream to, say, a time-binned histogram, or as required. For example in high throughput FLIM, when using the highly parallel multi-channel capability of the system to increase the conventional TCSPC limit, the only valuable information is the time between laser pulse and fluorescence photon event. For FCS, only the time between successive events need be measured. In both cases knowledge of the channel ID is not required. In both cases, the FPGA will be programmed to filter only the relevant data, which can then be transmitted to a data acquisition PC via the USB interface.

## 5 Conclusions

The IRPICS system, with its resolution of ∼25 ps and up to 1024 channels read out in parallel at rates of up to 10 MCount/s, will exploit the state-of-the-art event timing potential of small pore microchannel plates. It will offer unequaled combination of photon time resolution, channel density and event throughput, enabling measurements to be speeded up by orders of magnitude for applications such as fluorescence lifetime imaging.

The manufacture of the complete IRPICS system is close to completion. However delays in manufacture of the detector and electronic boards mean we do not have a complete IRPICS system with which to demonstrate performance. We have already achieved encouraging results with the 64 channel prototype detector [8]. A best result of 78 ps rms photon timing resolution was measured using a pulsed laser, which included broadening due to 65 ps rms laser trigger jitter and 50 ps laser pulse width.

We expect the integrated IRPICS design to outperform the prototype whose performance was comprised due to a distributed electronics design. Initial electronic tests carried out on the modular TDC board supports this. We have measured the time jitter between channels of the TDC board using an electronically generated signal at <22 ps rms.

The 32×32 pixel^2^ MLC has been manufactured and we are currently attaching the detector interface flange, followed shortly by detector assembly. The TDC board, backplane and FPGA digital processing board have all been manufactured and tested. All other electronic board PCBs have been designed and manufactured and are now being populated ready for test. We expect full IRPICS system testing to begin in the first quarter of 2012.
